# Incidence of infections due to third generation cephalosporin-resistant *Enterobacteriaceae* - a prospective multicentre cohort study in six German university hospitals

**DOI:** 10.1186/s13756-018-0452-8

**Published:** 2018-12-27

**Authors:** Anna M. Rohde, Janine Zweigner, Miriam Wiese-Posselt, Frank Schwab, Michael Behnke, Axel Kola, Birgit Obermann, Johannes K.-M. Knobloch, Susanne Feihl, Christiane Querbach, Friedemann Gebhardt, Alexander Mischnik, Vera Ihle, Wiebke Schröder, Sabina Armean, Silke Peter, Evelina Tacconelli, Axel Hamprecht, Harald Seifert, Maria J. G. T. Vehreschild, Winfried V. Kern, Petra Gastmeier, on behalf of the DZIF-ATHOS study group, Michael Buhl, Dirk Busch, Simone Eisenbeis, Gesche Först, Federico Foschi, Meyke Gillis, Dorothea Hansen, Georg Häcker, Markus Heim, Martin Hug, Klaus Kaier, Fabian Küpper, Georg Langebartels, Andrea Liekweg, Hans-Peter Lipp, Nayana Märtin, Mathias Nordmann, Andrea Pelzer, Luis-Alberto Peña-Diaz, Jan Rupp, Christin Schröder, Katrin Spohn, Michaela Steib-Bauert, Jörg J. Vehreschild, Ulrich vor dem Esche, Solvy Wolke

**Affiliations:** 1 German Center for Infection Research (DZIF), Inhoffenstraße 7, 38124 Braunschweig, Germany; 2 Charité – Universitätsmedizin Berlin, Institute of Hygiene and Environmental Medicine, Hindenburgstraße 27, 12203 Berlin, Germany; 3 Department of Infection Control and Hygiene, University Hospital Cologne, Kerpener Straße 62, 50937 Köln, Germany; 4 Department of Infectious Diseases and Microbiology, Institute for Medical Microbiology, University Hospital Schleswig-Holstein, Ratzeburger Allee 160, 23562 Lübeck, Germany; 5 Institute for Medical Microbiology, Immunology and Hygiene, Klinikum Rechts der Isar, Technische Universität München, Munich, Germany; 6 Division of Infectious Diseases, Department of Medicine II, Medical Center and Faculty of Medicine, University of Freiburg, Hugstetter Strasse 55, 79106 Freiburg, Germany; 7 Division of Infectious Diseases, Department of Internal Medicine 1, University Hospital Tübingen, Otfried-Müller-Straße 12, 72076 Tübingen, Germany; 8 Institute for Medical Microbiology and Hygiene, University Hospital Tübingen, Elfriede-Aulhorn-Straße 6, 72076 Tübingen, Germany; 9 Institute for Medical Microbiology, Immunology and Hygiene, University Hospital Cologne, Goldenfelsstrasse 19-21, 50935 Köln, Germany; 10 Department I of Internal Medicine, University Hospital of Cologne, Herderstraße 52-54, 50931 Köln, Germany; 11 Institute for Medical Microbiology, Virology and Hygiene, University Medical Center Hamburg-Eppendorf, Martinistraße 52, 20246 Hamburg, Germany

**Keywords:** Fluoroquinolone, Carbapenem, Gram-negative, ESBL, *Enterobacter* spp., *Klebsiella* spp., *E. coli*, CRE, Hospital-acquired infections, Community-acquired infections

## Abstract

**Background:**

Infections caused by third generation cephalosporin-resistant *Enterobacteriaceae* (3GCREB) are an increasing healthcare problem. We aim to describe the 3GCREB infection incidence and compare it to prevalence upon admission. In addition, we aim to describe infections caused by 3GCREB, which are also carbapenem resistant (CRE).

**Methods:**

In 2014–2015, we performed prospective 3GCREB surveillance in clinically relevant patient specimens (screening specimens excluded). Infections counted as hospital-acquired (HAI) when the 3GCREB was detected after the third day following admission, otherwise as community-acquired infection (CAI).

**Results:**

Of 578,420 hospitalized patients under surveillance, 3367 had a 3GCREB infection (0.58%). We observed a similar 3GCREB CAI and HAI incidence (0.28 and 0.31 per 100 patients, respectively). The most frequent pathogen was 3GCR *E. coli*, in CAI and HAI (0.15 and 0.12 per 100 patients). We observed a CRE CAI incidence of 0.006 and a HAI incidence of 0.008 per 100 patients (0.014 per 1000 patient days).

**Conclusions:**

Comparing the known 3GCREB admission prevalence of the participating hospitals (9.5%) with the percentage of patients with a 3GCREB infection (0.58%), we conclude the prevalence of 3GCREB in university hospitals to be about 16 times higher than suggested when only patients with 3GCREB infections are considered. Moreover, we find the HAI and CAI incidence caused by CRE in Germany to be relatively low.

## Background

Emerging multidrug-resistant Gram-negative bacteria are a global health concern, especially those harbouring extended-spectrum beta-lactamases (ESBL), which render *Enterobacteriaceae* resistant to third generation cephalosporins (3GC) and extended-spectrum penicillins [1]. Third generation cephalosporin resistant *Enterobacteriaceae* (3GCREB) infections are a special threat to patient safety, as resistance may cause a delay in effective antimicrobial therapy and thereby lead to worsening patient outcomes [2]. EU surveillance data shows that the 3GC resistance rate of *E. coli* in blood and cerebrospinal fluid samples has increased in many EU countries (EU mean 2012: 11.9%, 2015: 13.1%) [3]. The incidence density of 3GCREB in clinical specimens in German intensive care units (ICUs) rose from 2001 to 2015 (*E. coli*: 0.16 to 3.83/1000 patient days, *K. pneumoniae*: 0.25 to 1.41/1000 patient days) [4]. The percentage of hospital-acquired infections (HAI) caused by ESBL-producing *Enterobacteriaceae* in German ICUs and surgical departments increased as well (2007: 10.9% to 2012: 15.5%) [5].

The ATHOS (Antibiotic Therapy Optimization Study) project aimed at assessing the 3GCREB admission prevalence and 3GCREB incidence of community-acquired and hospital-acquired infections (CAI, HAI) in six German university hospitals in 2014 and 2015. The prevalence data was published previously [6]. Here, we describe the incidence of 3GCREB infections in the same hospitals and relate the data to the 3GCREB admission prevalence. Furthermore, we analyse the distribution of additional resistance phenotype patterns in those 3GCREB that caused infections.

## Methods

### Study design and data sources

The ATHOS project was a prospective observational cohort study that monitored hospitalized patients in general wards and ICUs for their first 3GCREB detection in clinical specimens (active surveillance). Each microbiology finding was followed by checking the health record or by contacting the wards and clinicians directly. The study was performed in six German university hospitals from January 1, 2014 to December 31, 2015. Patients hospitalized in the departments of dermatology, gynaecology/obstetrics, ophthalmology, otorhinolaryngology, paediatrics and psychiatry were excluded from surveillance.

### Microbiological analysis

Gram-negative bacteria were identified down to species level using either MALDI-TOF MS or VITEK®2 (bioMérieux, Nürtingen, Germany). Antimicrobial susceptibility testing was performed using VITEK®2. *Enterobacteriaceae* were classified as susceptible or resistant based on minimal inhibitory concentrations according to EUCAST breakpoints [7]. Non-susceptibility was regarded as resistance. Indicator antimicrobials for third generation cephalosporin resistance were cefotaxime and ceftazidime, indicators for carbapenem resistance were imipenem and meropenem.

### Definitions

A 3GCREB case was defined as the first 3GCREB isolate detected in clinical specimens (e.g. urine, wound swab, blood culture, tracheobronchial secretion or other clinical specimens) in a patient during a single hospital stay. Readmission followed by another 3GCREB detection created a new case.. Cases were distinguished in colonisations and cases with infections. Infections were defined as the detection of 3GCREB in a clinical specimen with additional signs and symptoms of infection as determined by a clinician followed by adequate antimicrobial therapy. A single case could present several 3GCREB species, each with several infections (though counting only by infection type). Acquisition was defined as follows: detection on day 1–3 (admission day = day 1) counted as community-acquired (CA), later detections counted as hospital-acquired (HA) [8]. We stratified for the following infection types: urinary tract infection (UTI), lower respiratory tract infection (LRTI), surgical site infection (SSI) and bloodstream infection (BSI). Other infection types were pooled in the category “other infections”. We analysed HAI caused by HA-3GCREB and CAI caused by CA-3GCREB. Cases with an ambiguous acquisition (CA-3GCREB with HAI and HA-3GCREB on top of existing CAI) were discarded.

### Statistical analysis

Infection incidence was calculated as infections per 100 patients, incidence density as infections per 1000 patient days. Both were stratified by species, resistance phenotype and infection type. Then 95% confidence intervals were calculated. The species distribution over infection types was tested with Χ^2^ test (R x C table). The comparison of resistance phenotypes among 3GCREB responsible for different infection types was tested with Fisher’s exact test (2 × 2 table, carbapenem-resistant versus -susceptible). *P*-values < 0.05 were considered significant. Statistical analysis was performed with SAS 9.4 (SAS Institute, Cary, NC, USA) and OpenEpi (Open Source Epidemiologic Statistics for Public Health, V3.01 http://www.openepi.com).

### Ethics and data protection

3GCREB surveillance was performed in accordance with the German Infection Protection Act [9]. The ethics committee at Charité, University Medicine Berlin, Germany, approved this study (EA/018/14). Data from the six hospitals was entered into an online accessible database approved by the data protection commissioner.

## Results

The ATHOS project was conducted at six German university hospitals comprising a total of 283 wards and 4957 beds in the surveillance area. The majority were general wards with surgical specialty (*n* = 104) followed by medical specialty (*n* = 83), intensive care units (ICU, *n* = 49), haematology/oncology (*n* = 35) and intermediate care wards (*n* = 12).

**Fig. 1:**
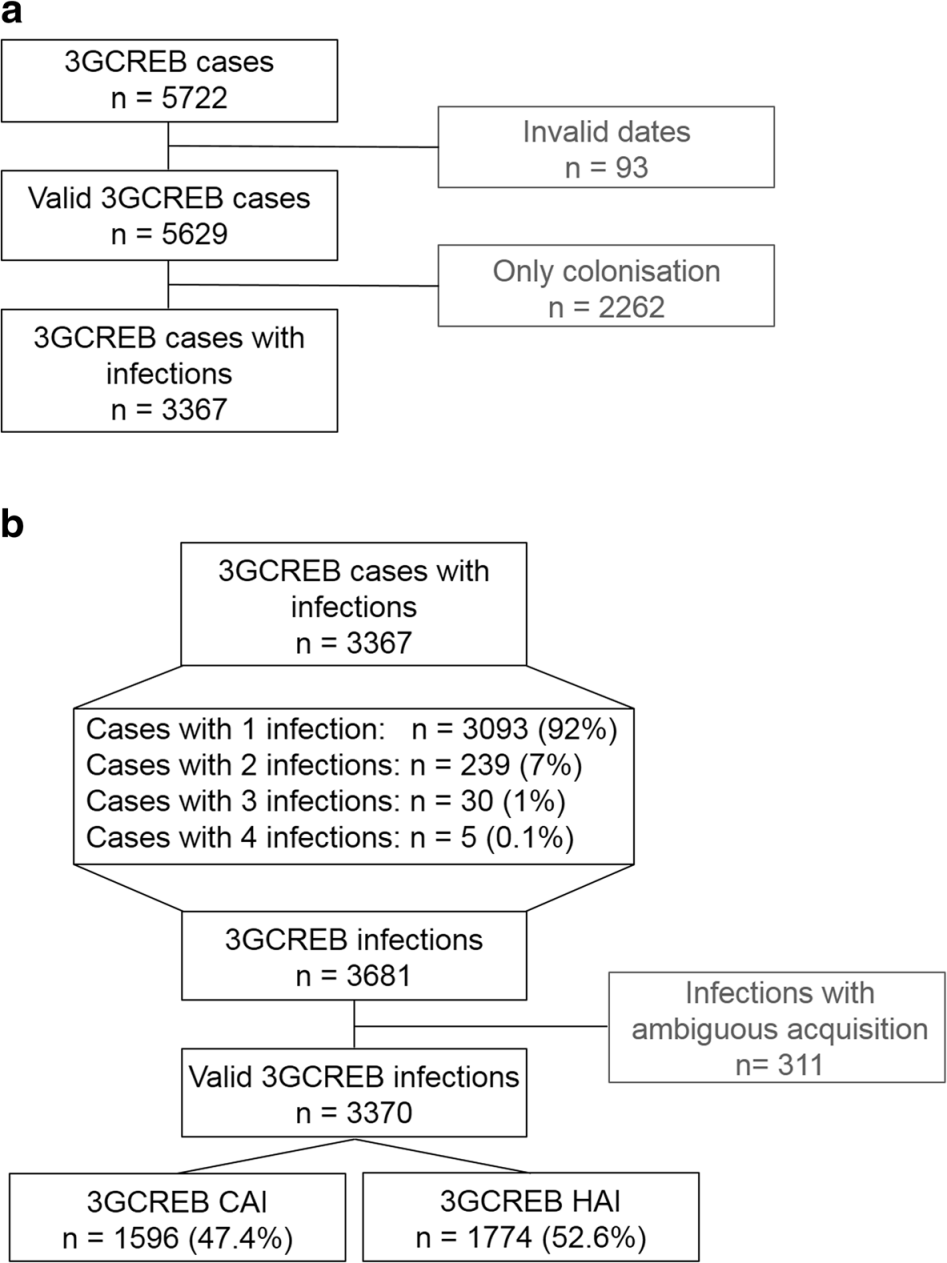
Flow chart of third generation cephalosporin resistant *Enterobacteriaceae* (3GCREB) cases (**a**) and infections (**b**). **a**) Readmission followed by 3GCREB detection created a second case. **b**) Each case could represent several infections but only one per infection type

In the period 2014–2015, 578,420 patient admissions with 3,385,112 patient days were under surveillance. After excluding invalid data and cases of colonization (*n* = 2262), 3367 clinical cases with one or more 3GCREB infections (0.58% of the patients) were analysed (Fig. 1a). The median age was 69 (IQR 58–77 years) and 55% of the cases were male. Of the cases, 92% had one infection, 7% had two infections and 1% had three or four infections.

To compare different infection types, cases were broken down into single infections and those with ambiguous acquisition were discarded, yielding 3370 single infections. The majority of infections were hospital-acquired (3GCREB HAI, Fig. 1b). We observed a difference in infection incidence among the university hospitals, e.g. the 3GCREB HAI incidence ranged from 0.17–0.42 per 100 patients. Therefore, the data of the individual hospitals was pooled for further analysis. The absolute numbers of infections stratified by species, resistance phenotype, and infection type are shown in Table 1, the infection incidences and incidence densities in Table 2. The 3GCREB CAI incidence was 0.28 per 100 patients, and that of HAI 0.31 per 100 patients. The majority of CAI were caused by 3GCREB which were also fluoroquinolone resistant (FQR, 0.17 per 100 patients), in HAI that incidence was lower (0.14 per 100 patients). 3GCREB that were also carbapenem resistant caused 0.006 CAI and 0.008 HAI per 100 patients.

**Table 1:** Distribution of third generation cephalosporin resistant *Enterobacteriaceae* (3GCREB) infections in the ATHOS project, 2014–2015, Germany

Parameter	Category	3GCREB infections *n* (%)	3GCREB CAI *n* (%)	3GCREB HAI *n* (%)
3GCREB	total	3370 (100%)	1596 (100%)	1774 (100%)
Species	*E. coli*	1624 (48%)	910 (57%)	714 (40%)
	*Enterobacter* spp.	702 (21%)	221 (14%)	481 (27%)
	*Klebsiella* spp.	562 (17%)	264 (17%)	298 (17%)
	*Citrobacter* spp.	209 (6%)	76 (5%)	133 (7%)
	Other species ^a^	273 (8%)	125 (8%)	148 (8%)
Resistance	all 3GCREB			
	only 3GCR	1488 (44%)	580 (36%)	908 (51%)
	+ FQR	1796 (53%)	979 (61%)	817 (46%)
	+ CR	86 (3%)	37 (2%)	49 (3%)
	*E. coli*	1624 (100%)	910 (100%)	714 (100%)
	only 3GCR	409 (25%)	209 (23%)	200 (28%)
	+ FQR	1203 (74%)	698 (77%)	505 (71%)
	+ CR	12 (1%)	3 (0%)	9 (1%)
	*Enterobacter spp.*	702 (100%)	221 (100%)	481 (100%)
	only 3GCR	553 (79%)	168 (76%)	385 (80%)
	+ FQR	122 (17%)	45 (20%)	77 (16%)
	+ CR	27 (4%)	8 (4%)	19 (4%)
	*Klebsiella spp.*	562 (100%)	264 (100%)	298 (100%)
	only 3GCR	157 (28%)	59 (22%)	98 (33%)
	+ FQR	362 (64%)	181 (69%)	181 (61%)
	+ CR	43 (8%)	24 (9%)	19 (6%)
Infections	SSI	401 (12%)	99 (6%)	302 (17%)
	UTI	1528 (45%)	872 (55%)	656 (37%)
	LRTI	571 (17%)	158 (10%)	413 (23%)
	BSI	459 (14%)	220 (14%)	239 (13%)
	Other infections ^b^	411 (12%)	247 (15%)	164 (9%)
Year	2014	1784 (53%)	847 (53%)	937 (53%)
	2015	1586 (47%)	749 (47%)	837 (47%)
Ward type	General ward	2174 (65%)	1102 (69%)	1072 (60%)
	ICU/interm. Care	1196 (35%)	494 (31%)	702 (40%)
Specialty	Surgical	1407 (42%)	522 (33%)	885 (50%)
	Non-surgical	1963 (58%)	1074 (67%)	889 (50%)

3GCREB infections among 578,420 patient admissions and 3,385,112 patient days in six German university hospitals*.* ^a^ “Other *Enterobacteriaceae* species” include *Cedecea, Hafnia, Morganella, Pantoea*, *Proteus, Providencia, Raoultella,* and *Serratia* species. ^b^ “Other infections” includes all other infection types. 3GCREB = third generation cephalosporin-resistant *Enterobacteriaceae*, *3GCR* third generation cephalosporin resistance, *FQR* fluoroquinolone resistance, *CR* carbapenem resistance, *CA* community-acquired, *HA* hospital-acquired, *SSI* surgical site infection, *UTI* urinary tract infection, *LRTI* lower respiratory tract infection, *BSI* bloodstream infection, *ICU* intensive care unit, *interm. Care* intermediate care. Column percentages were calculated for each parameter with respect to “3GCREB total”

**Table 2:** Incidence (densities) of infections with third generation cephalosporin-resistant *Enterobacteriaceae* (3GCREB), ATHOS project, 2014–2015, Germany

Parameter	Category	3GCREB CAI incidence per 100,000 admissions (95% CI)	3GCREB CAI incidence per 100 admissions (95% CI)	3GCREB HAI incidence per 100 admissions (95% CI)	3GCREB HAI incidence density per 1000 patient days (95% CI)
3GCREB	total	276 (263–290)	0.28 (0.26–0.29)	0.31 (0.29–0.32)	0.52 (0.50–0.55)
Species	*E. coli*	157 (147–168)	0.16 (0.15–0.17)	0.12 (0.12–0.13)	0.21 (0.20–0.23)
	*Enterobacter* spp.	38 (33–44)	0.04 (0.03–0.04)	0.08 (0.08–0.09)	0.14 (0.13–0.16)
	*Klebsiella* spp.	45 (40–52)	0.05 (0.04–0.05)	0.05 (0.05–0.06)	0.09 (0.08–0.10)
	*Citrobacter* spp.	13 (10–16)	0.01 (0.01–0.02)	0.02 (0.02–0.03)	0.04 (0.03–0.05)
	Other species ^a^	22 (18–26)	0.02 (0.02–0.03)	0.03 (0.02–0.03)	0.04 (0.04–0.05)
Resistance	all 3GCREB				
	only 3GCR	100 (92–109)	0.10 (0.09–0.11)	0.16 (0.15–0.17)	0.27 (0.25–0.29)
	+ FQR	169 (159–180)	0.17 (0.16–0.18)	0.14 (0.13–0.15)	0.24 (0.23–0.26)
	+ CR	6 (5–9)	0.006 (0.005–0.009)	0.008 (0.006–0.011)	0.014 (0.011–0.019)
	*E. coli*				
	only 3GCR	36 (31–41)	0.04 (0.03–0.04)	0.04 (0.03–0.04)	0.06 (0.05–0.07)
	+ FQR	121 (112–130)	0.12 (0.11–0.13)	0.09 (0.08–0.10)	0.15 (0.14–0.16)
	+ CR	1 (0–2)	0.001 (0.000–0.002)	0.002 (0.001–0.003)	0.003 (0.001–0.005)
	*Enterobacter spp.*				
	only 3GCR	29 (25–34)	0.03 (0.03–0.03)	0.07 (0.06–0.07)	0.11 (0.10–0.13)
	+ FQR	8 (6–10)	0.01 (0.01–0.01)	0.01 (0.01–0.02)	0.02 (0.02–0.03)
	+ CR	1 (1–3)	0.001 (0.001–0.003)	0.003 (0.002–0.005)	0.006 (0.003–0.009)
	*Klebsiella spp.*				
	only 3GCR	10 (8–13)	0.01 (0.01–0.01)	0.02 (0.01–0.02)	0.03 (0.02–0.04)
	+ FQR	31 (27–36)	0.03 (0.03–0.04)	0.03 (0.03–0.04)	0.05 (0.05–0.06)
	+ CR	4 (3–6)	0.004 (0.003–0.006)	0.003 (0.002–0.005)	0.006 (0.003–0.009)
Infections	SSI	17 (14–21)	0.02 (0.01–0.02)	0.05 (0.05–0.06)	0.09 (0.08–0.10)
	UTI	151 (141–161)	0.15 (0.14–0.16)	0.11 (0.11–0.12)	0.19 (0.18–0.21)
	LRTI	27 (23–32)	0.03 (0.02–0.03)	0.07 (0.07–0.08)	0.12 (0.11–0.13)
	BSI	38 (33–43)	0.04 (0.03–0.04)	0.04 (0.04–0.05)	0.07 (0.06–0.08)
	Other infections ^b^	43 (38–48)	0.04 (0.04–0.05)	0.03 (0.02–0.03)	0.05 (0.04–0.06)

^a^ “Other *Enterobacteriaceae* species” include *Cedecea, Hafnia, Morganella, Pantoea*, *Proteus, Providencia, Raoultella,* and *Serratia* species. ^b^ “Other infections” includes all other infection types. 3GCREB = third generation cephalosporin-resistant *Enterobacteriaceae*, *3GCR* third generation cephalosporin resistance, *FQR* fluoroquinolone resistance, *CR* carbapenem resistance, *3GCR + CR* carbapenem resistant *Enterobacteriaceae* (CRE), *CAI* community-acquired infection, *HAI* hospital-acquired infection, *SSI* surgical site infection, *UTI* urinary tract infection, *LRTI* lower respiratory tract infection, *BSI* bloodstream infection, *95% CI* 95% confidence interval

Among 3GCREB species, *E. coli* caused the highest incidence of CAI (0.15 per 100 patients) and HAI (0.12 per 100 patients). The most frequent infections were UTIs irrespective of the acquisition; the incidence of 3GCREB CA-UTI exceeded that of 3GCREB HA-UTI (0.15 vs. 0.11 per 100 patients). For SSI and LRTI, the incidence of 3GCREB HAI exceeded that of CAI (Table 2).

**Fig. 2:**
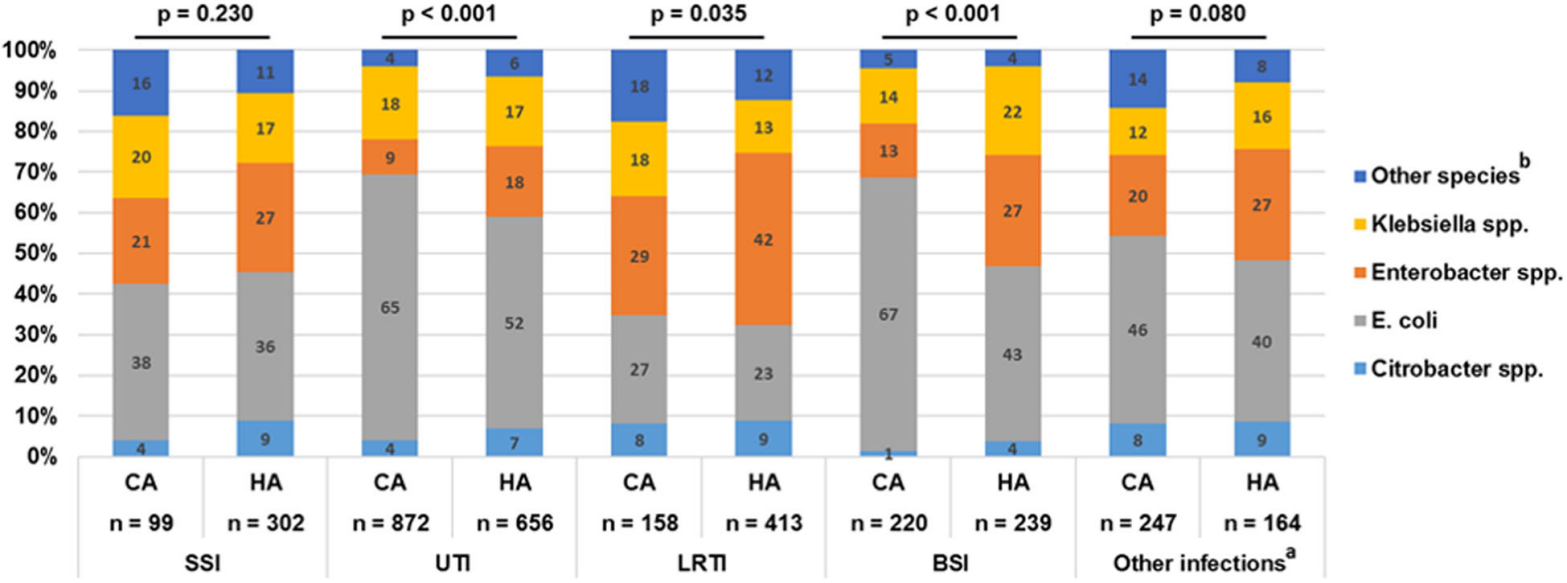
Relative species distribution in third generation cephalosporin-resistant *Enterobacteriaceae* (3GCREB) infections*,* stratified for infection type and acquisition, ATHOS project, 2014–2015, Germany. ^*a*^ *“Other infections” includes all infection types other than: SSI = surgical site infection, UTI = urinary tract infection, LRTI = lower respiratory tract infection, BSI = bloodstream infection. CA = community-acquired, HA = hospital-acquired.* ^*b*^ *“Other Enterobacteriaceae species” include Cedecea, Hafnia, Morganella, Pantoea, Proteus, Providencia, Raoultella, and Serratia species. Χ2-tests (5 × 2 table) were performed for each infection type to test for differences between CAI and HAI*

**Fig. 3:**
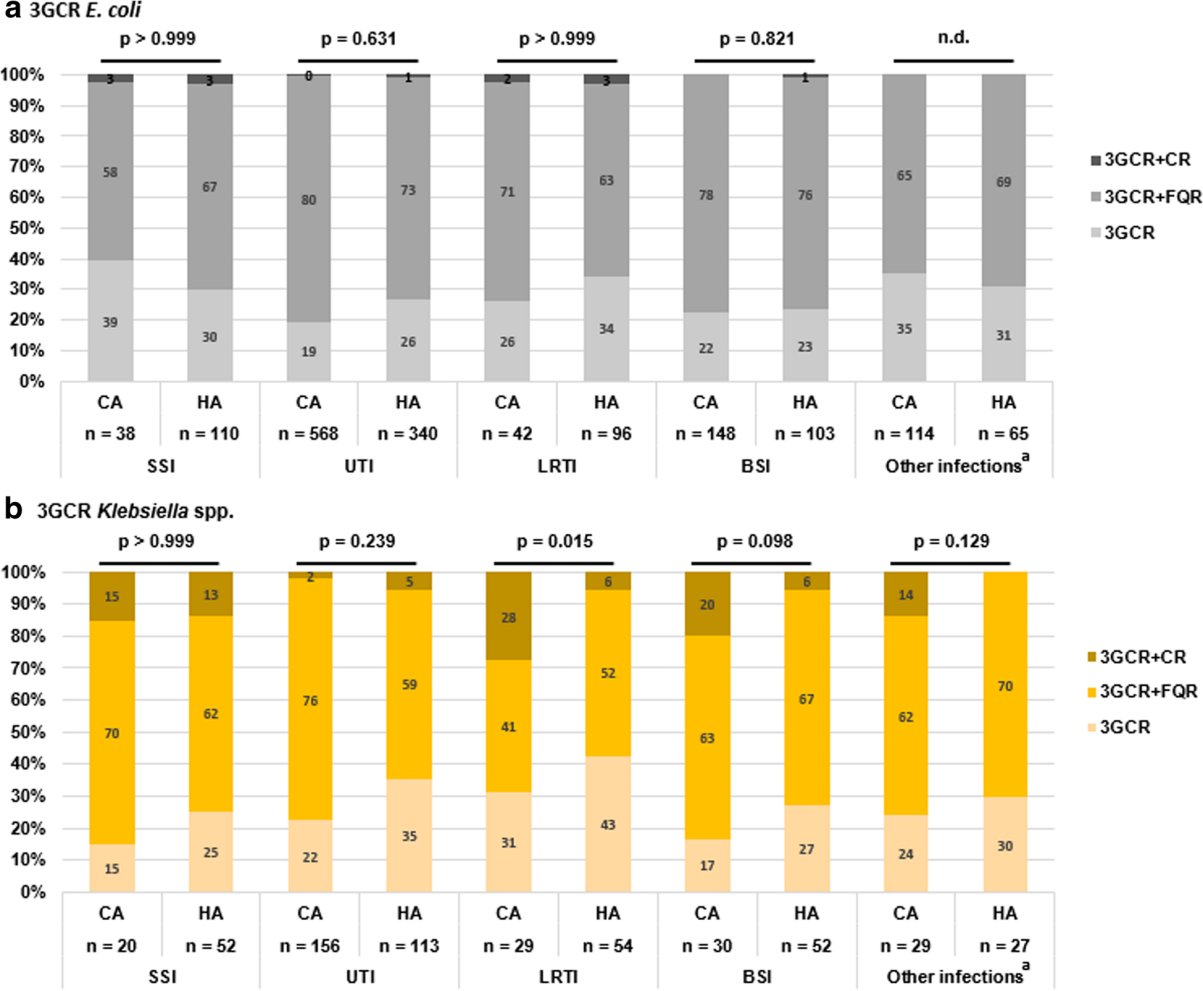
Relative distribution of resistance phenotypes of third generation cephalosporin-resistant (3GCR) *E. coli* (**a**) and *Klebsiella* spp. (**b**) depending on infection type and acquisition, ATHOS project, 2014–2015, Germany. ^*a*^ *“Other infections” includes all infection types other than: SSI = surgical site infection, UTI = urinary tract infection, LRTI = lower respiratory tract infection, BSI = bloodstream infection. 3GCR = third generation cephalosporin resistance, FQR = fluoroquinolone resistance, CR = carbapenem resistance, 3GCR + CR = carbapenem-resistant Enterobacteriaceae (CRE), CA = community-acquired, HA = hospital-acquired. Fishers exact-tests (2 × 2 table, carbapenem-resistant* versus *-susceptible) were performed for each infection type to test for differences between CAI and HAI, p < 0.05 was considered significant. “n.d.” = not defined, p-value cannot be calculated due to missing events in both groups*

Figure 2 shows the distribution of 3GCREB infections stratified by CAI/HAI and by species. Species distribution differed significantly in UTI, LRTI and BSI: HA-UTI, -LRTI and -BSI were caused more frequently by *Enterobacter* spp.. HA-BSI were also caused to a higher percentage by *Klebsiella* spp. than CA-BSI. Figure 3 shows the distribution of resistance phenotypes by infection type. The resistance phenotypes of 3GCR *E. coli* isolates did not differ between CAI and HAI (Fig. 3a). In contrast, 3GCR *Klebsiella* spp. showed a higher proportion of carbapenem resistance in all CAI except UTI. Twenty percent of the 3GCR *Klebsiella* spp. that caused CA-BSI and 28% of those that caused CA-LRTI were carbapenem resistant. A high percentage of 3GCR *Klebsiella* spp. that caused surgical site infections were also carbapenem resistant, irrespective of the acquisition (15% in CA-SSI and 13% in HA-SSI) (Fig. 3b).

## Discussion

The incidence of 3GCREB infections among patients admitted to German university hospitals in 2014/15 was < 1%. Additional fluoroquinolone resistance was frequent in particular in CAI, while additional carbapenem resistance was rare, both in CAI and HAI (0.006 and 0.008 per 100 patients; HAI incidence density 0.014 per 1000 patient days). An interesting finding was that among CA-LRTI caused by 3GCR *Klebsiella* spp.*,* the percentage of additional carbapenem resistance (28%) was substantial and significantly higher than in 3GCR *Klebsiella* spp. causing HA-LRTI (6%, *p* = 0.015).

In an admission prevalence study performed in parallel, we screened a minimum of 500 patients per hospital for rectal 3GCREB carriage on admission (day 1–3, admission day = day 1). This study showed a 3GCREB colonization prevalence of 9.5% [6]. Therefore, we conclude that the colonization rate of patients in university hospitals is about 16 times higher (=9.5%/0.58% patients with 3GCREB infection) than suggested when only patients with 3GCREB infections are considered. The reason for this high colonization prevalence and for a CAI incidence comparable to HAI is most likely the case mix in tertiary care university hospitals due to their position at the end of the treatment chain. On admission, many patients most likely have received previous antimicrobial treatment and have higher co-morbidity scores than patients in other hospital types.

The incidence of CAI causing 3GCREB that were also resistant to fluoroquinolones exceeded that of HAI (Table 2). One reason for this may be the high antibiotic use reported by patients admitted to the participating hospitals (34% of the 3GCREB-negative and 53% of the 3GCREB-positive patients) [6]. Another reason may be an enhanced use of fluoroquinolones in outpatient care [10]. This excess of fluoroquinolone use might be caused (among other reasons) by over prescription and non-adherence to antibiotic prescription guidelines in outpatient care [11–13]. A reduction of fluoroquinolone prescriptions would be desirable.

In 2013/14, the EU mean of carbapenemase-producing (CP) *E. coli* and *K. pneumoniae* was found to be 0.025 per 1000 patient days and 0.006 per 1000 patient days were reported for Germany (EuSCAPE study) [14]. We observed a CRE HAI incidence density for *Klebsiella* spp. of 0.006 (95% CI 0.000–0.008) and for *E. coli* of 0.003 per 1000 patient days (95% CI 0.001–0.005). The EuSCAPE study showed that among CRE, 70% of the *K. pneumoniae* and 30% of the *E. coli* produced carbapenemases [14]. Combining these percentages of CPE among CRE with our CRE data yields an estimated CPE incidence density for *Klebsiella* spp*.* and *E. coli* of 0.005 per 1000 patient days (*Klebsiella* spp. 0.004 and *E. coli* 0.001 per 1000 patient days). Thus, our data is comparable to the EuSCAPE data for Germany [14].

In contrast to other *Enterobacteriaceae* species, 3GCR *Klebsiella* spp. showed a high percentage of carbapenem resistance among CA infections, especially in LRTI and BSI. In a UK study, the prevalence of carbapenem resistance in clinically relevant *K. pneumoniae* specimens was also due primarily to community-acquired isolates (70%) [15]. The EU mean of carbapenem resistance in invasive *K. pneumonia*e isolates increased from 2012 to 2015 to 8%. In two European countries, carbapenem resistance was observed in over 25% of *K. pneumoniae* (Italy 34% and Greece 62%). In isolates from Germany, carbapenem resistance was rare (0.1%) [3]. Surveillance in German ICUs showed that the carbapenem resistance rate of *K. pneumoniae* in clinically relevant specimens increased from 2001 to 2015 to 1.5% [4]. A large admission prevalence study found a low CRE admission prevalence in Germany (comparable to UK, both 0.1%) [6, 16]. In light of this data, we conclude that even with increasing trends Germany currently still is a low CRE prevalence region.

The ATHOS project was a prospective observational study. One major limitation is the lack of patient-based information on previous healthcare contacts. Therefore, a classification into the important “healthcare-associated” category was not possible. Instead, the somewhat arbitrary day 3 limit for classification into CA and HA was applied, as it is commonly used for surveillance of HAI [8]. Nonetheless, we have some insight into the healthcare contacts of our patient mix from our patients in the admission prevalence study sample, 9% of whom had stayed in a rehabilitation centre, 5% in a long-term care facility and 26% in another hospital in the 6 months prior to admission [6]. Some CAI may, in fact, be healthcare-associated and thus the incidence of CAI may be overestimated in our analysis. This CA/HA classificiation was also used for SSI. We cannot exclude that some CA-SSI cases may be readmitted cases. An advantage of the study is the inclusion of general wards. We can describe the incidence of 3GCREB infections in German university hospitals comprehensively and are not limited to ICU data. However, due to the hospital-wide surveillance, we lack ward-specific denominator data and are not able to calculate department-specific incidences.

One strength of the study is the inclusion of most *Enterobacteriaceae* species, since other studies are often restricted to *E. coli* or *K. pneumoniae.* Such studies are likely to underestimate the real incidence of CRE. We found a major part of CRE-HAI caused by *Enterobacter* spp. (0.006 per 1000 patient days, 95% CI 0.000–0.008). Imipenem resistance of *E. cloacae* complex has increased over the last years in German ICUs [4]. In addition, long-term surveillance by the US Veterans Health Administration of CRE in patients also showed a steady increase of carbapenem resistance in *E. cloacae* [17]. However, we have noticed that Vitek®2 frequently overreported imipenem resistance. For future CRE studies, we suggest including *Enterobacter* spp. and using additional, more reliable diagnostic methods (e.g. disk diffusion test or agar gradient diffusion) to determine if the high frequency of Vitek®2 imipenem resistance represents the true epidemiology. Another drawback of using routine diagnostic methods is that we were unable to compare sequence types of 3GCREB causing CAI and HAI.

As a result of the 3GCREB admission prevalence study performed in parallel [6] and the surveillance data for infections, we can for the first time estimate the 3GCREB prevalence to be about 16-times higher than indicated by the 3GCREB infection incidence (0.58%). This will enable other university hospitals with a similar patient mix to estimate roughly the dimension of colonization prevalence present in their patients. Furthermore, we conclude that very few 3GCREB carriers are identified using clinically indicated diagnostic procedures. Therefore, we believe the majority of hospitals underestimate the extent of 3GCREB prevalence. This study gives a comprehensive description of the incidence of CRE found in German university hospitals.

## Conclusion

Overall, our analysis showed that German university hospitals have a low 3GCREB infection incidence when compared to the admission prevalence. As we observed a comparable incidence of 3GCREB CAI and HAI, it is important that clinicians consider cephalosporin resistance in their empirical treatment decisions, irrespective of the acquisition type (CA vs. HA) of the infection.

## Abbreviations

3GC (3GCR): 3rd generation cephalosporin (−resistant)
3GCREB: 3rd generation cephalosporin-resistant *Enterobacteriaceae*
ATHOS: Antibiotic therapy optimisation study
BSI: Bloodstream infection
CA (CAI): Community-acquired (infection)
CP (CPE): Carbapenemase-producing (*Enterobacteriaceae*)
CR (CRE): Carbapenemase-resistant (*Enterobacteriaceae*)
*E. cloacae*: *Enterobacter cloacae*
*E. coli*: *Escherichia coli*
ESBL: extended spectrum β-lactamase
EU: European Union
EUCAST: European committee on antimicrobial susceptibility testing
HA (HAI): Hospital-acquired (infection)
ICU: Intensive care unit
IQR: Interquartile range
*K. pneumonia*: *Klebsiella pneumonia*
LRTI: Lower respiratory tract infection
MALDI-TOF MS: Matrix-assisted laser desorption/ionization-time of flight mass spectrometry
spp.: species
SSI: Surgical site infection
UK: United Kingdom
US: United States
UTI: Urinary tract infection
